# Farcimab: a flicker of light in the darkness of neovascular age-related macular degeneration and Diabetes Mellitus – correspondence

**DOI:** 10.1097/JS9.0000000000000281

**Published:** 2023-03-14

**Authors:** Omer Ahmed Shaikh, Shifa Amin, Gulrukh Shaikh, Kanchan Kumari, Irfan Ullah, Muhammad Sohaib Asghar

**Affiliations:** a Department of Medicine, Ziauddin University; b Department of Medicine, Liaquat National Hospital and Medical College; c Jinnah Sindh Medical University, Karachi; d Kabir Medical College, Gandhara University, Peshawar, Pakistan; e Division of Nephrology and Hypertension, Mayo Clinic, Rochester, Minnesota, USA

*Dear Editor*,

Age-related macular degeneration (AMD) is ‘an acquired degeneration of the retina due to the modification of the Bruch’s membrane and the retinal pigment epithelium’^1^. AMD can be classified as either atrophic (dry form), present in 80% of the patients, or exudative/neovascular (wet form), affecting up to 15% patients^1^,^2^. Although less prevalent, neovascular age-related macular degeneration (nAMD) accounts for 90% of subsequential vision loss, with patients affected by the dry form also eventually progressing to nAMD^1^. Diabetic macular edema (DME), is one of the main manifestations of diabetic retinopathy^3^. Both DME and nAMD are associated with overexpression of vascular endothelial growth factor-A (VEGF-A), particularly in the vitreous and retina, causing alteration in the genes responsible for angiogenesis and vascular permeability, with consequential breaking of the blood retinal barrier and accumulation of fluid in the macula^1^,^3^,^4^. With the global number of patients with AMD estimated to rise to up to 288 million by 2040 and DME expected to cause visual impairment in up to 25% of patients with diabetic retinopathy in the USA by 2030, it is safe to assume the two may easily be classified as the most common contributors to ocular morbidity worldwide^1^–^3^.

The current FDA-approved treatment for both nAMD and DME is intravitreal anti-VEGF injections, including Aflibercept, Ranibizumab, Bevacizumab, and Brolucizumab, which inhibit neovascular proliferation^4^. However, with multiple clinical trials, it has been observed that 68% of nAMD patients on anti-VEGF do not achieve the threshold for driving vision (best-corrected visual acuity of 20/40 Snellen equivalent) despite a year of treatment; even increasing the dose frequency has not shown any increase in efficacy or durability of results^5^. Furthermore, the high-frequency treatment, requiring multiple visits and injections, along with the unsatisfactory outcome and vision loss over time, have contributed to lapses in care, especially in DME patients^4^. One possible hypothesis for these results is that multiple pathways contribute to choroidal neovascularization, one of them being the angiopoietin-Tie2 (Ang/Tie2) axis^4^,^5^. This pathway complements VEGF-mediated activity in retinovascular diseases by Ang-2 upregulation, resulting in vascular leakage, pericyte loss, and inflammation^4^. Since anti-VEGF therapy alone has not provided the desired outcome, combining it with Ang-Tie targeted drugs may help reduce the disease burden.

A recent study by Khanani *et al*.^4^ suggests that Faricimab, a bi-specific intravitreal antibody that simultaneously binds and neutralizes both Ang-2 and VEGF-A could be a turning point in the treatment of both DME and nAMD. Multiple randomized controlled trials have been conducted to find the efficacy and safety of the drug, along with comparing it to the currently available treatment. Successful phase 1 and phase 2 studies, including BOULEVARD for DME and AVENUE and STAIRWAY for nAMD, have confirmed not only patient safety and tolerance for the drug but also improved best-corrected visual acuity and the diabetic retinopathy severity score^6^. Since Faricimab is still in phase 3 trials, little to no information is available regarding its adverse effects. However, concern has been raised regarding the use of anti-VEGF while breast-feeding. The presence of VEGF in breast milk is believed to aid in gastrointestinal tract maturation. Although unlikely due to its large size, small amounts of Faricimab may be present in breast milk; therefore, lactating women are advised to observe caution until more data becomes available^7^.

Since both the diseases are highly prevalent and major liabilities to healthcare worldwide, it is imperative that we take prompt action to reduce not only the disease burden but also the treatment burden. Bi-specific treatment like Faricimab will result in more efficient treatment with fewer dose requirements, reducing the number of visits and therefore decreasing the overall treatment burden on the patient and provider, both in terms of cost and time. This may promote long-term adherence to the treatment, which might eventually improve the overall visual outcomes for both nAMD and DME. Faricimab may thus be a valuable contribution to the ophthalmological community. Therefore, it is imperative that further studies be promptly conducted, as this would undoubtedly be a positive development.

## Ethical approval

Not applicable as no patient data involved.

## Consent

Written informed consent not applicable as no study participants are involved.

## Sources of funding

There are no sources of funding.

## Author contribution

O.A.S. and S.A. conceived the idea. G.S. and K.K. performed a literature review and wrote the manuscript. M.S.A. and I.U. reviewed the manuscript and critically revised it to the final form. All authors approved the final version of the manuscript.

## Conflicts of interest disclosure

The authors declare no conflict of interest.

## Research registration unique identifying number (UIN)

1. Name of the registry: NA.
2. Unique identifying number or registration ID: NA.
3. Hyperlink to your specific registration (must be publicly accessible and will be checked): NA.

## Guarantor

Muhammad Sohaib Asghar.

## Data sharing statement

Not applicable.

## Provenance and Peer review

Externally peer reviewed, not commissioned.
